# Characteristics and Stability of Mercury Vapor Adsorption over Two Kinds of Modified Semicoke

**DOI:** 10.1155/2014/260141

**Published:** 2014-08-27

**Authors:** Zhang Huawei, Liu Xiuli, Wang Li, Liang Peng

**Affiliations:** College of Chemical and Environmental Engineering, Shandong University of Science and Technology, Qingdao, Shandong Province 266510, China

## Abstract

In an attempt to produce effective and lower price gaseous Hg^0^ adsorbents, two methods of HCl and KMnO_4_/heat treatment were used respectively for the surface modification of liginite semicoke from inner Mongolia. The different effects of modification process on the surface physical and chemical properties were analyzed. The characteristics and stability of mercury vapor adsorption over two kinds of modified semicoke were investigated. The results indicated that modification process caused lower micropore quantity and volume capacity of semicoke; the C-Cl functional groups, C=O bond and delocalized electron *π* on the surface of Cl-SC, the amorphous higher valency Mn^*x+*^, and O=C–OH functional groups on the surface of Mn-H-SC were the active sites for oxidation and adsorption of gaseous Hg^0^. Modification process led to higher mercury removal efficiency of semicoke at 140°C and reduced the stability of adsorbed mercury of semicoke in simulated water circumstance simultaneously.

## 1. Introduction

Gaseous Hg^0^ pollutant in flue gas has the properties of being stable, thermodynamic, and insoluble in water, and its removal techniques have become a research focus. At present, one of the most effective ways for gaseous Hg^0^ removal is injecting varieties of solid adsorbents into flue gas, which can oxidize the elemental mercury into Hg^2+^ and capture it [1]. Previous research shows that high efficiency gaseous Hg^0^ adsorbents can be prepared using cheap porous materials by physical and chemical surface modification methods [2–6]. Lignite semicoke has similar chemical properties with activated carbon, but its price is only one-third of activated carbon or so, which can be widely used as low-cost adsorbent in the fields of flue gas desulfurization and denitrification, oil purification, and wastewater treatment [7–9]. However, there are seldom reports about semicoke as mercury adsorbents of flue gas; the well-developed pore structure and abundant functional groups on the surface of semicoke can provide active sites for the removal of gaseous Hg^0^ [10]. It is expected to prepare high efficiency and cheap gaseous Hg^0^ adsorbents by surface modification of lignite semicoke.

For the mercury adsorbed semicoke, it must be disposed as dangerous goods due to its high mercury content and potential toxicity; therefore, the mercury stability on the surface of adsorption product is the major factor which determines its subsequent treatment process. Indeed, in most of coal-fired power plants, the adsorbents injection device is always installed in the upstream of dust cleaning equipment, and the semicoke after mercury adsorption reaction would be captured together with fly ash in the dust cleaning unit. Fly ash is a kind of precious resource and can be used for the preparation of glass ceramics, cement, fertilizer, and sewage purification material [11, 12]. If the adsorbed mercury on the surface of semicoke is not stable, it will pollute the fly ash product. In the subsequent process of fly ash utilization, the adsorbed mercury is likely to reescape due to the influence of environmental temperature, humidity, and solution pH value and thus causes secondary pollution on the environment. Therefore, it is necessary to investigate the adsorbed mercury speciation and stability on the semicoke surface and provide reference data for follow-up treatment measure and process for the adsorption product.

## 2. Experimental 

### 2.1. Sample Preparation

The lignite semicoke used in the tests is from the Huolin River of Inner Mongolia; the preparation method is as follows: we placed a coal sample in a 700°C muffle furnace, conducted 1 h of destructive distillation under anoxic conditions, cooled it to room temperature, and then smashed it to 80 to 100 meshes for use and recorded it as NM-SC; we soaked 10 g of prepared NM-SC for 24 h into mass fractions 25% HCl solution, with a 1 : 3 of solid-liquid ratio; after the reactions were completed, we filtrated and dried the sample, and the semicoke treated with the HCl may be obtained, which was recorded as Cl-SC; NM-SC was impregnated in a potassium permanganate solution (0.06 mol/L), stirred for 4 hour at 90°C in a water bath, filtered, and dried, and we took thermal treatment of impregnated sample at 260°C under N_2_ protection for 1.5 hour and recorded it as Mn-H-SC. After the mercury adsorption experiments for 20 h, the samples of Cl-SC and Mn-H-SC were named Hg-Cl-SC and Hg-Mn-H-SC.

### 2.2. Characterization

The physical characterizations of the samples were analyzed by the Brunauer-Emmett-Teller (BET) method, and the surface area and pore size distribution were obtained by adsorbing and desorbing in N_2_ at 77 K, using an automatic volumetric multipoint apparatus (SSA-4300). Before the measurements were taken, all of the samples were outgassed at 100°C for 2 h. The microstructures of the samples were characterized by scanning electron microscopy (KYKY2800B). X-ray photoelectron spectroscopy (Thermo ESCALAB250) with Al K*α* (*hν* = 1486.6 eV) as the excitation source was used to determine the binding energies of C1s, O1s, Mn2p, and Hg4f. The C1s line at 284.6 eV was taken as a reference for the binding energy calibration.

### 2.3. Mercury Adsorption Experimental Methodology and Instrumentation

A small fixed bed reactor described in [4] was employed to evaluate the gaseous Hg^0^ removal efficiency of semicoke under the simulated flue gas condition. The adsorption efficiency was calculated as follows:

(1) $$\begin{array}{c} \eta =1-\frac{C_{t}}{C_{0}}, \end{array}$$

where *η* indicates adsorption efficiency, *C*_0_ is the initial concentration of gaseous Hg^0^, and *C*_*t*_ indicates the concentration of gaseous Hg^0^ after adsorption. The mercury volume quantity in unit mass of adsorbent was calculated as follows:

(2) $$\begin{array}{c} q=\frac{C_{0}Lt-\int_{0}^{t}CL\;dt}{m}, \end{array}$$

where *q* represents mercury volume quantity in unit mass of adsorbent, *L*is the gas flow, *t* is the adsorption time, and *m* is the dosage of adsorbent.

Mercury concentration in flue gas was measured online by QM201 mercury analyzer, in which detection range is from 0 to 50 *μ*g/m^3^, and mercury concentration in leaching solution was measured by SG-921 mercury analyzer, in which detection range is from 0.1 to 10 ng/mL.

### 2.4. The Toxicity Characteristic Leaching Procedure and Sequential Chemical Extraction Experiments of Adsorption Products

The toxicity characteristic leaching procedure (TCLP) tests are carried out according to the methods in [13]; we weighed 0.5 g adsorption sample accurately, put it in glacial acetic acid solution whose pH value is 4.93, where the mass ratio of solid to liquid is 1 : 20, impregnated it for 20 h, and determined the mercury concentration in the supernatant. The details of sequential chemical extraction experiments are given in [14, 15]; the semicoke samples of adsorption products were extracted using the following solutions: (1) 1 mol/L MgC1_2_, (2) 10 mol/L HCI, (3) 2 mol/L HNO_3_, (4) 50 g/L Na_2_S in l0 g/L NaOH, and (5) aqua regia. The corresponding mercury forms in extraction for each step are as follows: (1) water soluble, (2) exchangeable, (3) hydrochloric acid soluble, (4) nitric acid soluble, and (5) mercuric sulfide and residual mercury.

## 3. Results and Discussion

### 3.1. Analysis of Specific Surface Area and Pore Structure

From the data shown in Table 1, the specific surface area and pore structure analysis of three kinds of semicoke, it can be seen that the surface area and total pore volume were slightly increased after HCl treatment; this is because HCl can react with ash in semicoke and produce soluble chloride; part of channels blocked by ash are got through in the following washing process and cause the surface area and total pore volume to increase. However, the HCI treatment only increased the volumes of the mesopores and macropores; in the meantime, it also gave rise to the partial transition of the micropores to mesopores and macropores; this conclusion is consistent with previous research of Dubinin [16] and Yanxu et al. [17]. After KMnO_4_/heat treatment, the specific surface area of semicoke decreased, the proportion and volume of micropores obviously reduced, and the average pore size increased, which indicated that part of micropore structures were destroyed during the KMnO_4_/heat treatment process.

### 3.2. Analysis of Scanning Electron Microscopy (SEM)

**Figure 1:**
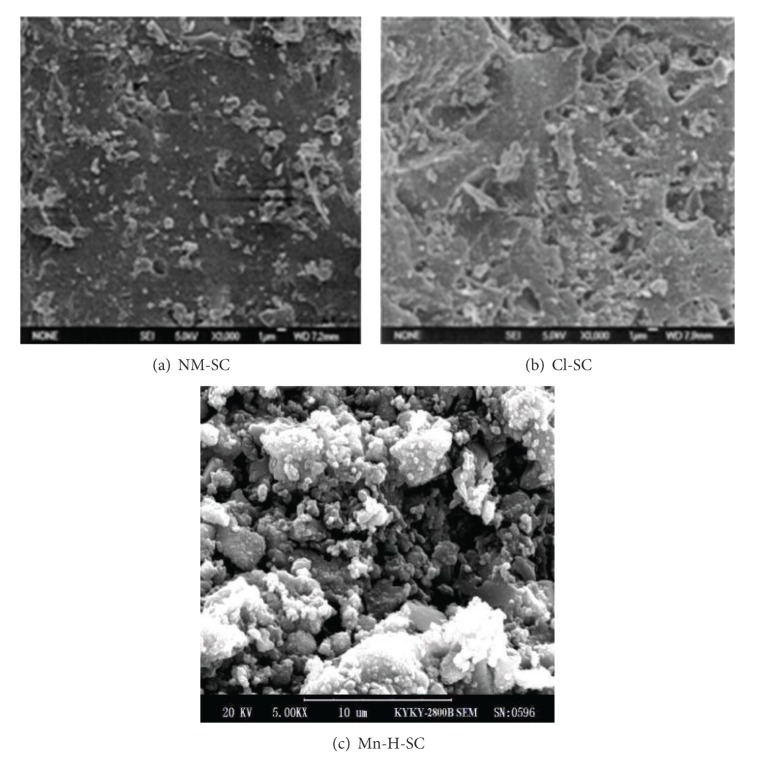
SEM images of NM-SC, Cl-SC, and Mn-H-SC.

Figure 1 describes the surface microtopography of NM-SC, Cl-SC, and Mn-H-SC. It can be seen that the surface of crude semicoke is flat, and the pores on the sample have the properties of uneven size and irregular shape. HCI treatment did not destroy the pore structure of crude semicoke but clearly generated many new pores with different sizes. After KMnO_4_/heat treatment, the surface of Mn-H-SC was covered by a layer of attachment; parts of pores were blocked up and caused the surface roughness of the samples to increase significantly.

### 3.3. Analysis of X-Ray Photoelectron Spectroscopy (XPS)

X-ray photoelectron spectroscopy (XPS) was applied to analyze the surface oxygenic functional groups of three kinds of adsorbents (NM-SC, Cl-SC, and Mn-H-SC), two kinds of adsorbents with captured gaseous Hg^0^ (Hg-Cl-SC and Hg-Mn-H-SC), and valence state of manganese oxide of two samples (Mn-H-SC and Hg-Mn-H-SC). The narrow region spectrum of C1s and Mn2p_3/2_ was fitted by multipeaks using XPS Peak Processing software. The fitting parameters of C1s are C–C–C (284.0~285.1 eV), C–OR (285.3~287.0 eV), C=O (286.8~288.1 eV), and O=C–OH (288.1~290.0 eV) [18], and those of Mn2p_3/2_ are Mn^7+^ (645.6 eV), Mn^6+^ (644.2 eV), Mn^4+^ (643.0 eV), Mn^3+^ (642.1 eV), and Mn^2+^ (641.0 eV) [19, 20].

According to the fitting results of C1s spectrum shown in Table 2, it may be concluded that C–C carbon species are the main carbon components on the surface of SC (51.37%), followed by the carbon species C–OR (34.32%), C=O (9.48%), and O=C–OH (4.83%). HCl modification increased the carbon species of C–C, C=O, and O=C–OH by 15.59%, 1.74%, and 4.20%, respectively, and reduced the carbon species of C–OR by 21.53%. After KMnO_4_/heat treatment, the carbon species of C–C and O=C–OH on semicoke increased by 23.80% and 2.04% and C–OR and C=O decreased by 20.92% and 4.92%, respectively. In general, two kinds of modification methods can increase graphitization degree, the content of carboxylic acid and ester chemical functional groups of semicoke. HCl treatment also increased the content of alcohols, ethers, ketones, quinones, and other chemical functional groups, and KMnO_4_/heat treatment reduced the content of those functional groups. For Cl-SC and Mn-H-SC samples, surface graphite carbon content increased slightly after gaseous Hg^0^ adsorption, while the carbon content of carbonyl or quinone functional groups and carboxyl or ester functional groups decreased slightly; it is probably due to the fact that, in the gaseous Hg^0^ adsorption process, part of C=O groups of ketones, quinones, carboxylic acids, and esters oxidized gaseous Hg^0^ and at the same time it was reduced, causing the C=O double bond to be broken, thus resulting in the decrease of carbonyl or quinone functional groups and carboxyl or ester functional groups of semicoke.

The Mn2p_3/2_ multipeak fitting results of Mn-H-SC and its adsorption product are shown in Table 3. It can be seen that the average oxidation degree of manganese on the surface of Mn-SC is 3.46; there is no Mn^7+^ compounds. These results indicated that the potassium permanganate decomposed during the modification, and a portion was reduced to a lower valence state by the surface organic functional groups or carbon atoms of semicoke [21, 22]. After gaseous Hg^0^ adsorption at 140°C, compared with Mn-H-SC, the average manganese oxidation state on Hg-Mn-H-SC surface reduced by 0.38, Mn^7+^ spectrum peak disappeared, the atomic ratio of Mn^4+^ decreased slightly, and the atomic ratio of Mn^3+^ and Mn^2+^ rose sharply. This showed that Mn^6+^ and Mn^4+^ on the surface of Mn-H-SC would oxidize gaseous Hg^0^ to Hg^2+^ in adsorption process and were reduced to their lower valence state, Mn^3+^and Mn^2+^.

### 3.4. Gaseous Hg^0^ Adsorption Characteristics of Semicoke

**Figure 2:**
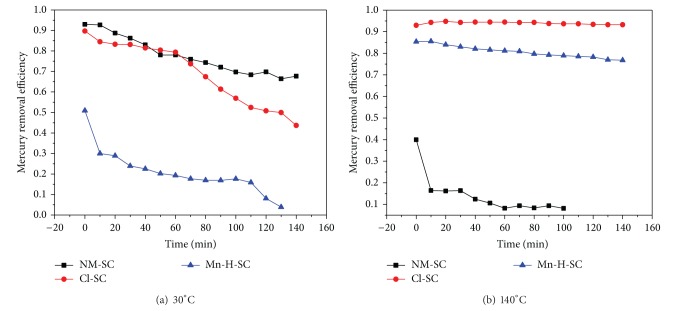
Mercury removal efficiency of NM-SC, Cl-SC, and Mn-H-SC at 30°C and 140°C.

In Figure 2, under the conditions that the inlet mercury concentration is 30 *μ*g/m^3^, gas flow is 1 L/min, adsorbent is 0.50 g, and adsorbent temperature is 30°C and 140°C, the gaseous Hg^0^ removal efficiency of NM-SC, Cl-SC, and Mn-H-SC is described in Figure 2.

It is observed that when the adsorption temperature is 30°C, NM-SC has a good mercury removal performance. At low temperature, gaseous Hg^0^ takes physical adsorption as the primary reaction on the semicoke surface; the gaseous Hg^0^ is primarily absorbed in the micropores, and the surface area and pore structure of semicoke are the principal factors that determine the mercury removal performance. The analysis data of the specific surface area and pore structure showed that the crude semicoke had a relatively large specific surface area and pore volume, and the surface micropore proportion reached to 72.4%; the average pore diameter was 2.55 nm; thus it has high mercury removal efficiency. The BET analysis data showed that although HCl treatment increased the total pore volume and surface area of semicoke slightly, the proportion and volume of micropore declined and the physical adsorption capacity of semicoke to gaseous Hg^0^ reduced to a certain extent, thereby leading to Cl-SC that presented lower mercury removal efficiency. KMnO_4_/heat treatment decreased mercury removal efficiency of semicoke at 30°C obviously; it is due to the fact that a portion of pores on semicoke surface is clogged by attachments after modification process, and heat treatment at 260°C causes partial collapse of micropores, which led to part transition of micropores to mesopores, so the micropore portion and volume of Mn-H-SC were significantly lower than NM-SC and performed poor mercury removal performance at 30°C.

When the adsorption temperature is 140°C, the mercury removal efficiency of Cl-SC was much higher than that of NM-SC and was maintained above 90% after 140 min adsorption reaction. Gaseous Hg^0^ mainly takes chemical adsorption on semicoke surface at high temperature; HCl treatment has great influence on surface chemical properties of semicoke, mainly to increase the contents of the functional groups such as carboxyl and the phenolic hydroxyl groups, and in the modification process, the Cl^−^ in hydrochloric acid solutioncantake ion-exchange reaction on the semicoke surface, thus enabling part of chlorine elements to be absorbed on the semicoke surface and forming the Cl–C–Cl functional groups. When the concentration of the Cl^−^ on the semicoke surface is high, the gaseous Hg^0^ can react with Cl^−^ and produce mercury chlorides, such as [HgCl]^+^, HgCl_2_, or [HgCl_4_]^2−^ [23, 24]. The C=O bond and delocalized electron *π* on the semicoke surface also may be taken as electron acceptors, thus enabling the reaction between Hg^0^ and Cl^−^ to be conducted towards the positive direction and generating the stable chloride of mercury. Both the oxygen-containing functional groups and chlorine-containing groups on Cl-SC surface can provide active sites for gaseous Hg^0^ oxidation and chemical adsorption, thus remarkably increasing the mercury removal efficiency of Cl-SC at 140°C. The mercury removal efficiency of Mn-H-SC was slightly lower than Cl-SC but significantly higher than NM-SC. KMnO_4_/heat treatment modification can increase the content of O=C–OH groups in carboxyl and ester groups on semicoke surface and generate the amorphous MnO_*x*_ compounds with higher valence; the average oxidization degree of manganese is 3.46. Gaseous Hg^0^ can be oxidized to Hg^2+^ by high valence Mn^*x*+^ and O=C–OH functional groups and chemically adsorbed in the pore channels of Mn-H-SC, making the mercury removal performance improve significantly.

### 3.5. Mercury Speciation and Stability on the Surface of Semicoke

For adsorption products, the adsorbed mercury stability in simulated water environment was investigated using TCLP leaching tests, and the mercury speciation on the surface of semicokes was studied using sequential chemical extraction experiments.

The TCLP leaching experiment results of three kinds of semicoke samples are shown in Table 4. It can be seen from the TCLP tests data that the mercury stability of semicoke samples after adsorption reaction has great relationship on the adsorption temperatures and modification methods of semicoke. For the samples of 30°C mercury adsorption products, no mercury was detected in the leachate of NM-SC samples; mercury concentrations in the leachate of Cl-SC and Mn-H-SC were 7.6 *μ*g/L and 9.0 *μ*g/L, respectively, which are lower than the safe concentration of TCLP standard value of 25 *μ*g/L. The stability of adsorbed mercury in micropores of semicoke is relativity better at low temperatures and is not easy to escape in the leaching process. The micropore proportion of Cl-SC and Mn-H-SC samples is lower than that of NM-SC, so causing the mercury stability on the surface of semicoke to somewhat decrease. For the samples of 140°C mercury adsorption products, the mercury concentration in the filtrate of Cl-SC sample was the highest and reached 168.2 *μ*g/L; the mercury concentration in the filtrate of Mn-H-SC sample was higher than that of NM-SC. At higher adsorption temperature, gaseous Hg^0^ can be oxidized to HgCl_2_ and HgO, respectively, on the surface of Cl-SC and Mn-H-SC. The solubility of elemental mercury, HgO, and HgCl_2_ is 0.06, 53, and 6.9 × 10^4^ mg/L, respectively; HgCl_2_ is easily soluble in water, which makes the mercury concentration in the filtrate of Cl-SC sample significantly high; HgO has lower solubility in water, so the mercury concentration in the filtrate of Mn-H-SC sample is below the safety standard value. In short, the modification process improved the mercury removal performance of semicoke at high temperatures but in different extent reduced the stability of adsorbed mercury on the surface of semicoke in simulated water environment.

The sequential chemical extraction experiment results of semicoke adsorption products are shown in Table 5. The experiment error of total mercury content is less than 10%, which indicated the reliability of the sequential chemical extraction. In the sequential extraction process, the extract of the first step is water soluble mercury; the exchangeable mercury of the second step mainly refers to the organic mercury which is instable on the surface of semicoke; the hydrochloric acid dissolved mercury of the third step means the HgO generated by the reaction of gaseous Hg^0^ and active oxidization in semicoke such as manganese oxides MnO_*x*_ and amorphous and crystalline Fe, Al oxides; the nitrate dissolved mercury of the fourth step refers to the elemental mercury adsorbed on the semicoke surface; the extract of the fifth and sixth steps is HgS and residual mercury, respectively [15, 25]. For the mercury adsorption products of NM-SC, Cl-SC, and Mn-H-SC at 30°C and 140°C, the content of HgS and residual mercury was about 10%, which may be HgS partially contained in semicoke itself or the stable forms of mercury generated in the reaction of gaseous Hg^0^ and sulfur or nitrogen element in semicoke.

Water soluble and exchangeable mercury were not detected in the extract of NM-SC adsorption product at 30°C, and 2.8% of hydrochloric acid solution mercury were detected, which is the HgO generated in the reaction of gaseous Hg^0^ and oxygen-containing functional groups or the amorphous and crystalline Fe, Al oxides in semicoke; the content of nitrate soluble mercury is as high as 89.6%, which is mainly the elemental mercury physical adsorbed on the semicoke surface. For the extract of NM-SC adsorption product at 140°C, hydrochloric acid soluble mercury content increased to 24.9% and nitric acid soluble mercury content reduced to 61.6%, indicating that the content of HgO increased, while the content of elemental mercury decreased in the sample of NM-SC adsorption product at high temperatures.

It could detect 79.7% of elemental mercury in the extract of Cl-SC adsorption product at 30°C and also contained a small amount of oxidation states and unstable organic mercury. For the adsorption product of 140°C, mercury mainly exists in water soluble form, and its content is 64.4%; this is because the gaseous Hg^0^ can take reaction with Cl element of semicoke and generate HgCl_2_ with high solubility. There was also part of exchangeable and acid soluble mercury on Cl-SC, mainly referring to unstable organic mercury or HgO generated in the reaction of gaseous Hg^0^ with surface oxygen-containing groups such as carboxyl, lactone, and hydroxyl.

The main mercury form of Mn-H-SC adsorption products at 30°C and 140°C was elemental and HgO, respectively.

## 4. Conclusions

1. Hydrochloric acid treatment mainly increased the volume of mesopores and macropores of semicoke and the content of chemical functional groups such as alcohols, ethers, ketones, and quinines on the semicoke surface. KMnO_4_/heat treatment combination modification process caused a decrease of micropore proportion on the semicoke surface, increased the graphitization degree and the content of carboxylic acid or ester chemical functional groups, and reduced the chemical functional groups content of alcohols, ethers, ketones, and quinines.
2. Gaseous Hg^0^ mainly took physical adsorption on the surface of semicoke at low temperature, while chemical adsorption was the main mechanism at high temperature; the Cl–C–Cl functional groups, C=O bonds and delocalized electrons *π* of Cl-SC, and the high valence Mn^*x*+^ and O=C–OH functional groups of Mn-H-SC could be taken as electron acceptors, made gaseous Hg^0^ oxidize to Hg^2+^, and chemically adsorbed in the pore channel of semicoke, thus improving the mercury removal efficiency of semicoke obviously.
3. The TCLP leaching experiment results indicated that the modification process could improve the mercury removal performance of semicoke at high temperatures but in different extent reduced the stability of adsorbed mercury on the surface of semicoke in simulated water environment. The contents of elemental mercury were the highest in three samples after 30°C adsorption, and the major adsorbed mercury forms of NM-SC, Mn-H-SC, and Cl-SC after 140°C adsorption were elemental HgO and HgCl_2_, respectively.

## Figures and Tables

**Table 1:** The BET surface area and pore parameters of semicoke.

Sample	BET surface area (m^2^/g)	Average pore size (nm)	Pore volume (m^3^/g)			Proportion %	
			Total	Micro (≤2 nm)	Meso	Micro (≤2 nm)	Meso
NM-SC	84.203	2.55	0.107	0.0775	0.0295	72.4	27.6
Cl-SC	97.278	2.96	0.113	0.0704	0.0426	62.3	37.7
Mn-H-SC	65.494	3.67	0.120	0.0602	0.0598	50.2	49.8

**Table 2:** Surface carbon functional groups distribution of NM-SC, Cl-SC, and Mn-H-SC.

Sample	C–C (%)	C–OR (%)	C=O (%)	O=C–OH (%)
SC	51.37	34.32	9.48	4.83
Cl-SC	66.96	12.79	11.22	9.03
Mn-H-SC	75.17	13.40	4.56	6.87
Hg-Cl-SC	77.18	12.69	5.98	4.15
Hg-Mn-H-SC	77.83	14.68	3.21	4.28

**Table 3:** Distribution of surface Mn ions of Mn-H-SC, and Hg-Mn-H-SC.

Sample	Mn^7+^ (%)	Mn^6+^ (%)	Mn^4+^ (%)	Mn^3+^ (%)	Mn^2+^ (%)	Mn AOS
Mn-H-SC	0.00	10.62	35.59	32.57	21.22	3.46
Hg-Mn-H-SC	0.00	0.00	32.02	44.10	23.88	3.08

AOS: average oxidation state.

**Table 4:** TCLP experiment results of three kinds of semicoke.

Sample	NM-SC		Cl-SC		Mn-H-SC	
	30°C	140°C	30°C	140°C	30°C	140°C
*μ*g/L	BDL	0.18	7.6	168.2	9.0	3.3

BDL: below detection limitation.

**Table 5:** Sequential extraction procedure results of semicoke adsorption products.

Sample	Water soluble (%)	Exchangeable (%)	HCl soluble (%)	HNO_3_ soluble (%)	HgS (%)	Residual (%)	Total (%)
NM-SC							
30°C	0	0	2.8	86.9	3.2	5.7	98.6
140°C	0	1.1	24.9	61.6	5.3	4.9	97.8
Cl-SC							
30°C	0.9	2.6	6.5	79.7	4.8	3.9	98.4
140°C	64.4	13.2	15.1	2.7	3.6	3.3	102.3
Mn-H-SC							
30°C	0	1.7	5.9	79.1	5.4	4.3	96.4
140°C	3.8	6.4	67.2	15.3	5.1	4.8	102.6
